# Identifying Barriers and Facilitators of 13 mHealth Projects in North America and Africa: Protocol for a 5-Year Implementation Science Study

**DOI:** 10.2196/resprot.9633

**Published:** 2018-07-03

**Authors:** Binyam Tilahun, Kirsten Smillie, Kevin Louis Bardosh, Melanie Murray, Mark Fitzgerald, Victoria Cook, Iraj Poureslami, Jamie Forrest, Richard Lester

**Affiliations:** ^1^ Division of Infectious Disease Faculty of Medicine University of British Columbia Vancouver, BC Canada; ^2^ Department of Health Informatics and eHealthLab Ethiopia University of Gondar Gondar Ethiopia; ^3^ Department of Anthropology University of Florida Gainesville, FL United States; ^4^ Division of Respiratory Medicine Faculty of Medicine University of British Columbia Vancouver, BC Canada; ^5^ Department of Tuberculosis Control British Columbia Centre for Disease Control Vancouver, BC Canada

**Keywords:** mobile health, mHealth, text messaging, digital health, implementation science, Africa, North America

## Abstract

**Background:**

Although many mHealth interventions have shown efficacy in research, few have been effectively implemented and sustained in real-world health system settings. Despite this programmatic gap, there is limited conclusive evidence identifying the factors that affect the implementation and successful integration of mHealth into a health system.

**Objective:**

The aim of this study is to examine the individual, organizational, and external level factors associated with the effective implementation of WelTel, an mHealth intervention designed to support outpatient medication adherence and engagement in care in Africa and North America.

**Methods:**

We will adopt the Consolidated Framework for Implementation Research (CFIR) constructs for evaluation of mHealth implementation including a scoring and monitoring system. We will apply the adapted tool to identify facilitators and barriers to implementation of the WelTel mHealth intervention in order to determine how the technology platform is perceived, diffused, adapted, and used by different mHealth project teams and health system actors in Africa and North America. We will use a mixed-methods approach to quantitatively test whether the factors identified in the CFIR framework are associated with the successful uptake of the mHealth intervention toward implementation goals. We will triangulate these data through interviews and focus group discussion with project stakeholders, exploring factors associated with successful implementation and sustainment of these interventions.

**Results:**

The development of the customized CFIR is finalized and currently is in pilot testing. The initial results of the use of the tool in those 13 implementations will be available in 2019. Continuous conference and peer- reviewed publications will be published in the coming years.

**Conclusions:**

The results of this study will provide an in-depth understanding of individual, organizational, and external level factors that influence the successful implementation of mHealth in different health systems and geographic contexts over time. Via the tool’s unique scoring system connected to qualitative descriptors, these data will inform the most critical implementation targets and contribute to the tailoring of strategies that will assist the health system in overcoming barriers to implementation, and ultimately, improve treatment adherence and engagement in care.

**Registered Report Identifier:**

RR1-10.2196/9633

## Introduction

### Background

Billions of dollars are globally spent on health research projects each year, but only a limited number of effective interventions are translated into practice and policy [1,2]. Technology is providing unprecedented opportunities to improve patient engagement in care for better adherence leading to reduced morbidity and mortality, yet a significant knowledge-action gap remains [2]. Many evidence-based innovations are not adequately scaled up to meet the full challenges of the United Nations’ global Sustainable Development Goals [1,3-5]. Mobile health (mHealth) is an emerging area of disease management that can help patients adhere to prolonged treatment regimens and improve their quality of care—an area where improvement can have more impact than even discovery of new treatments. Globally, mobile phones are now the most pervasive and accessible form of two-way communication technology. In 2014, the number of mobile phone subscriptions surpassed the number of people, and uptake in vulnerable and marginalized groups has, in many cases, bridged the socioeconomic “digital divide” [6]. Text messaging via short message service (SMS) remains one of the most popular forms of mobile communication and has become the most used data transfer system in the world, with over 24 billion text messages sent and received each day. A landmark randomized controlled trial (RCT), WelTel Kenya1, was the first comprehensive RCT to test effects of text message reminders in improving HIV therapy adherence. The authors showed that bi-directional, weekly text messaging significantly improved adherence to antiretroviral therapy (ART) and increased the proportion of patients with a suppressed HIV viral load in an HIV-positive Kenyan population initiating ART, over a 1-year period [7]. As noted by a recent Cochrane review, this RCT study (and many others) have built a strong body of high-quality evidence supporting the use of weekly text messaging to enhance ART adherence and viral suppression, in comparison to current standards of HIV/AIDS care [8].

Since the original RCT, the WelTel mHealth program has expanded in Africa and North America in the area of HIV/AIDS [9-12], tuberculosis (TB) [13], maternal and child care, asthma, and recently in primary care settings. The WelTel program is a patient-centered form of digital outreach communications designed to support engagement in care and treatment adherence. Using a weekly check-in model, usually via text message, patients self-identify concerns that are then triaged by a point person and connected for care to appropriate health care providers on an “as-needs” basis, leading to a form of patient-centered precision care. With its leading evidence base in HIV and TB, the WelTel program is ideal for an implementation science evaluation as it is in the process of being scaled-up in numerous settings.

Case studies, RCTs, and other quasi-experimental methods generate critically important evidence to determine the efficacy of an intervention. These methods, however, do not provide the necessary information required to implement comprehensive public health interventions in real-life settings. Implementation science can help fill this gap by studying the process of developing, introducing, institutionalizing, and sustaining policies, programs, and activities in complex settings. This is done by identifying the individual, population, health system, and health environment factors associated with the successful uptake of effective interventions [5,14-18].

Undertaking a comprehensive implementation science study is particularly important when studying mHealth interventions, as the health care system itself is complex, includes multiple interacting components, and interventions must be adapted to fit the needs of a practice setting and patients with different views and expectations [19,20]. However, to our knowledge there is no tested tool that can be continuously used to evaluate, monitor, and inform mHealth implementation processes as they unfold.

### Aim

The overarching aim of our 5-year research project is to create and test an implementation research tool that can be used to generate continuous, context-specific evidence that can inform mHealth for impact at scale. To do so, we will evaluate the scale-up process of the WelTel text-messaging mHealth system in different global implementation settings. We will use rigorous scientific methods to assess processes and outcomes with the following main objectives:

1. To adapt the Consolidated Framework for Implementation Research (CFIR) constructs for evaluation of mHealth implementation including a scoring and monitoring system
2. To apply the adapted tool to identify facilitators and barriers to implementation within each program to continuously inform the implementation process
3. To use a scoring framework to correlate implementation factors with measures of implementation success (rate and scale of adoption plus sustainability) across the multiple projects over time

### Conceptual Framework

This comprehensive mHealth implementation science study will use the widely cited and used CFIR [5]. The CFIR is an implementation science framework that provides a comprehensive taxonomy of operationally defined constructs from multiple disciplinary domains (eg, psychology, sociology, organizational change) that are likely to influence implementation of complex programs. CFIR constructs are organized into five major domains: (1) characteristics of the intervention (eg, evidence strength and quality, complexity), (2) the outer setting (eg, patient needs and resources), (3) inner setting (eg, compatibility of the mHealth intervention with existing engagement programs, leadership engagement), (4) characteristics of individuals involved (eg, knowledge and attitudes), and (5) the process used to implement the program (eg, quality and extent of planning, engagement of key stakeholders) [5]. We are using this framework with the primary aim of understanding how the WelTel mHealth program is perceived, diffused, adapted, and used by different mHealth project teams and health system actors in Africa and North America. The CFIR framework draws together the unique and common elements of 19 different theories and frameworks and offers a common taxonomy for exploring the effectiveness of implementation within a specific context [5]. While theoretical frameworks in implementation studies are underused [21], the use of theory in implementation studies can help identify factors that predict the likelihood of implementation success and help develop better strategies to achieve more successful implementation, thus strengthening the understanding and explanation of how and why implementation succeeds or fails (eg, what works, for whom, under what circumstances, and why) [22]. Theories, frameworks, and models can help identify appropriate outcomes, measures, and variables of interest for implementation studies. Theory can also help organize studies when collecting, analyzing, interpreting, explaining, and presenting data [23].

In preparation for this study, the appropriateness of several theories and frameworks were assessed. Several implementation frameworks and theories that exist were considered relevant, such as the Reach Effectiveness Adoption Implementation Maintenance framework [24], the Promoting Action on Research Implementation in Health Services framework [25], the Technology Acceptance Model [21], and the Normalization Process Theory [22]. The CFIR was chosen based on its comprehensiveness and ability to manage both breadth and depth of data given the complexity of mHealth programs. It includes a broad number of aspects related to implementation and is thus considered a helpful framework for identifying barriers and facilitators influencing mHealth implementation. It addresses the need to assess and maximize the effectiveness of implementation within a specific context and to promote dissemination to other contexts.

Since its inception, CFIR has largely been used to help understand the interplay between context and the implementation process. This framework can therefore be adapted and used to guide the design of mHealth interventions for particular settings, as well as the study of their implementation. An increasing number of implementation studies have used CFIR, some as an evaluation framework [23,26,27], some for detecting factors influencing implementation [28,29], and some for classifying these influencing factors as facilitators or barriers [19,26]. To date, only a few studies have employed the CFIR for evaluation of specific technological interventions [19,30,31]. We found only two other studies that used the CFIR developers’ method to identify and compare distinguishing constructs between high versus low implementation settings [19,32]. We were unable to find any studies that compared implementation factors in high- and low-resource settings. Finally, we did not find any studies that considered the dynamic nature of implementation by examining barriers and facilitators over time. [33-36]. There is a need for research that assesses, tests, and further develops CFIR’s applicability in determining which factors influence implementation success in the field of mHealth interventions. We will tailor the CFIR for use with mHealth interventions in different settings and contexts.

### Intervention Details

The WelTel innovation is an evidence-based, low-cost, easy-to-use, and accessible mobile phone‒based health communication solution to address the global challenge of inadequate outpatient engagement across multiple diseases. Developed with direct input from patients and caregivers in Kenya, the original WelTel model for HIV care was a weekly two-way text-message check-in by clinic staff to patients using basic mobile phones. Clinicians called patients if they reported a problem via text message, triaged those problems, and provided advice on how to manage them (eg, a care path). This method was first validated in the landmark RCT, WelTel Kenya1, which demonstrated improved patient adherence to HIV treatments and achieving viral suppression, a key target of the World Health Organization (WHO) and the Joint United Nations Programme on HIV and AIDS (UNAIDS) 90-90-90 target [7]. The mobile communication model was intended to provide patients with extra support between clinic visits, while maintaining maximal reach and privacy protection through its simplicity and lack of outgoing health-related content. It also extends the capacity of health care providers to look after patients by proactively managing outpatient problems and identifying individuals who require the most support (only 3% of patients identified “problems” each week). Multiple studies have since validated elements of the intervention, such as preferred message frequency (weekly better than daily) and the two-way nature of communication (versus one-way) [37]. The WelTel innovation, and evidence surrounding it, has directly informed global treatment guidelines such as the 2013 WHO ART guidelines and the 2014 International Association of Providers of AIDS Care guidelines for retention in HIV care. Regardless of its effectiveness, there are also different user and technological level challenges in different contexts. The WelTel service has been adapted for TB; maternal, neonatal, and child health; and asthma programs, with interest and the potential to expand to other health conditions in the future (eg, cancer care, primary care). In our adoption of WelTel for TB in British Columbia, Canada, we found that WelTel weekly two-way text messaging did not improve latent TB infection (LTBI) completion rates compared to standard LTBI care. However, completion rates were high in both treatment arms [38]. In a recent RCT to determine whether a text-messaging intervention improved retention during the first year of HIV care, WelTel’s weekly text-messaging service did not improve retention of people in early HIV care [39]. With all this mixed evidence and effectiveness across different implementations, we are proposing a continuous 5-year implementation science research on this intervention to identify what works and what does not.

## Methods

### Overview

In this cross-project, mixed-methods study, we will take an investigative approach to determining what works, what does not, and the specific barriers and facilitators to implementing the WelTel mHealth service at various programmatic stages. Using a standardized adapted CFIR tool, 13 initial projects will be evaluated at their current state and followed over time at 6-month intervals for up to 5 years. In addition to the adapted CFIR domain narratives, each domain will be scored using a standardized scoring technique (see below) and compared both across projects and longitudinally within projects. The scoring will be used to identify domains and elements within the domains that appear to be doing well (scored highly) or doing poorly (low scores), and how they are associated with successful progress in the implementation of the projects. These factors will then be discriminated in a way that can actively inform individual projects along the implementation process and ultimately lead to an optimized and cost-effective mHealth strengthening opportunity for these health services globally. The evidence from data collection every 6 months will be iteratively analyzed and used as an input to inform the implementation process. The results of each project will be shared with the respective project leaders and discussed in the bi-annual meetings of the research team on the modality of incorporating the evidence into the implementation process. Continuous support will be provided by the research team to see the level of incorporation of the research results into the implementation. Experience sharing visits will be also organized among different projects to facilitate evidence use among all 13 projects.

A novel feature of this study will be a cross-project implementation science evaluation using the CFIR framework. An international consortium for implementation science recognized the need to not only evaluate study endpoints but also conduct formative evaluations to assess the extent to which implementation is effective in a specific context to optimize an intervention’s benefits, improve sustainability, and promote dissemination of findings into other settings. The framework not only evaluates static components of implementation factors that facilitate or impede implementation success but also evaluates processes over time. The CFIR framework is flexible and was designed to be adaptable to a program’s specific needs. For our purposes, we will create a standardized scoring framework and narrative based on the most salient individual constructs of the framework for mHealth research and our specific intervention. This allows us to compare implementation factors across our individual studies in a standardized way and also evaluate them dynamically over time [40].

### Data Collection

Members of the mHealth research team will conduct one-on-one interviews or focus groups (depending on availability and preference of participants) using a purposive sampling strategy that focuses on individuals who are involved in the planning, implementation, or follow-up of their respective mHealth studies. Participants will consist of individual project relevant/representative stakeholders representing the range of stakeholders and representing the five domains. Five people from each project will be included in the qualitative study. To avoid bias, participants will be selected by each project leader rather than the central WelTel team. They will be asked to provide written informed consent. Interviews will be conducted at locations that are convenient for the key stakeholders and will ensure their privacy and confidentiality. Participants will have the opportunity to discuss potential strategies about how their respective project(s) could be improved or strengthened, the influence of internal and external social relationships and climate on the study and acceptance of the intervention, and suggestions for the WelTel mHealth intervention itself. Engaging with these team members through interviews or small focus groups will provide important insights that can guide implementation of the WelTel mHealth intervention in specific contexts. As each project team is at a different phase of their project, we will investigate difference stages of project development, implementation, and follow-up. Data will be collected every 6 months from each of the projects for 5 years.

### Scoring

**Figure 1:**
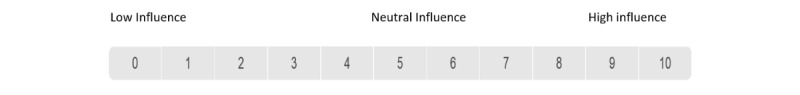
Scoring system that will be used to score each of the Consolidated Framework for Implementation Research (CFIR) constructs (adapted from the original CFIR authors).

Each of the constructs in the modified CFIR will have scoring out of 10. Each of the participants will score each of the constructs, and the median will be automatically calculated. A 10-point scoring approach will be used to flexibly capture the opinion of the study participants. A unique scoring guide will be used to maintain consistency among individual assessments and for standardized comparison across studies and over time. Figure 1, adapted from the original CFIR authors [5], shows the scoring system that will be used to score each of the CFIR constructs based on the level of influence they have on the implementation outcome.

### Participating Sites

This study is based on a coordinated set of 13 projects, operating on funding from different organizations in Canada, the United States, Kenya, Rwanda, South Africa, and Ethiopia. A cross-section of the 13 current projects will be followed, at regular intervals, as the studies are being completed. The study will take a pragmatic approach and include new projects during the envisioned path to scale and remove or replace projects that do not move forward after collecting all the important data from them regardless of the project outcome.

#### WelTel LTBI (British Columbia)

This is a dually funded project by the BC Lung Association and Canadian Institute of Health Research (CIHR). It evaluates the effect of WelTel on treatment completion among patients with LTBI in an RCT at two TB clinics in British Columbia (Vancouver and New Westminster). The study includes a cost-effectiveness evaluation and stakeholder assessment for health system integration (ClinicalTrials.gov identifier: NCT01549457).

#### WelTel Retain (Kenya)

This RCT (ClinicalTrials.gov identifier: NCT01630304) aims to assess whether the WelTel intervention improves retention in care in the first year of care after HIV diagnosis in economically disadvantaged cities in Nairobi. The study is funded by the National Institute of Mental Health.

#### WelTel at Oak Tree (British Columbia)

Following a successful pilot study of the intervention at the Oak Tree Clinic at BC Women’s Hospital, additional funding was secured by the Oak Tree team to recruit 100 HIV positive participants for a further evaluation involving clinical outcomes one year before and one year after implementation of the intervention, as well as its cost-effectiveness and health care provider time utilization (ClinicalTrials.gov identifier: NCT02603536).

#### The Cedar Project (British Columbia)

This project tests the feasibility, acceptability, and efficacy of using WelTel text messages to improve treatment adherence and resiliency for young First Nations people who are HIV-positive or at high risk of HIV. Project sites are in British Columbia and include Vancouver, Prince George, and Chase.

#### WelTel Big River (Saskatchewan)

The aim of this study is to understand the feasibility and acceptability of implementing the WelTel text messaging program with people living with HIV and hepatitis C in Big River First Nation, Saskatchewan, Canada. A general waiting-room survey will be offered to those who attend the Big River First Nation Health Facility to understand their digital technology use and their attitudes towards communicating with health care providers using these strategies. Pilot study participants will receive check-in messages once a week from an automated platform. The study team will measure if this increased engagement has an impact on the health of participants.

#### WelTel Kenya2 (Kenya)

This second phase “transition to scale” project, *Changing Global Health One Text at a Time*, is co-supported by Grand Challenges Canada and Amref Health Africa. The aim is to scale-up the WelTel intervention in Kenya’s vast northern and arid lands as part of a government-hosted consortium of health-strengthening initiatives, using the Integrated Innovation framework (social, technological, and business innovation). Currently there are five project sites: two in Isiolo County and three in Samburu County.

#### EmPhAsIS: Empowering Pharmacists in Asthma Management Through Interactive SMS (British Columbia)

This cluster-RCT at 75 pharmacies in British Columbia is designed to examine whether an adaptation of the WelTel intervention into pharmacy services improves adherence to asthma medication. The project site is in Vancouver.

#### Asthma Telehealth (British Columbia): WelTel eAsthma

The Asthma Telehealth project is a randomized trial testing the adaptation of the WelTel platform to link patients with moderate to severe asthma to their previously validated Asthma Action Plans, thus supporting outpatient self-management.

#### WelTel HIV (Seattle, USA)

This study is a pilot to assess WelTel in supporting high-needs HIV patients in Seattle and a second pilot for HIV pre-exposure prophylaxis adherence support. It is a National Institute of Health‒funded project that began in 2016.

#### WelTel Haida Gwaii (British Columbia)

This pilot implementation uses the WelTel platform and service to support primary care in the Queen Charlotte medical center. It will focus on supporting patient-oriented goals and will be linked to the Northern Health Authority electronic medical record system for remote communities.

#### WelTel Ethiopia (Gondar, Ethiopia)

This project is piloting implementation of the WelTel platform in clinical care to improve patient engagement in HIV care. The study is taking place at the ART unit of the University of Gondar hospital in rural northern Ethiopia.

#### WelTel Outreach (British Columbia)

This project is a scale-up program to assess the accessibility, feasibility, and transferability of the WelTel digital platform by the Outreach Team at the BC Centre for Disease Control after their successful use of WelTel LTBI for 3 years.

#### WelTel South Africa (South Africa)

The aim of this study is to examine the acceptability and feasibility of mHealth/SMS and community-based directly observed ART (cDOT) as interventions to improve ART adherence for preventing mother-to-child HIV transmission in a community primary care setting in Cape Town, South Africa.

### Participants

The main participants of this study will be stakeholders, individual project team members, and patients of the different implementations in global settings. Project team members who worked for more than 6 months will be eligible to be study participants to make sure they received enough exposure and experience about the project. For project participants, those who are older than 18 years of old will be included. As per our knowledge, most of the projects have an average of 5 team members to run the intervention. Hence, we target 5 participants for each stage of the assessment per project until saturation is reached.

#### Project Team Members

The team comprises individuals who are employed (eg, research coordinator, research assistant, statistician, epidemiologist, intern, data analyst), participating in (eg, clinicians who consent participants, respond to participants via the platform, clerical staff who administer questionnaires, operations manager who oversee staff duties) or advising and directing (ie, investigators, research fellows and associates, graduate students) the project.

#### Stakeholders

Individuals who have a stake in the success or failure of each project may include clinicians not directly involved in the operation of the study, as well as administrators, research participants, privacy and securities experts, government staff interested in mHealth technologies, staff at nongovernmental organizations, policymakers, donors, etc.

### Analysis

Our analytic approach will initially focus on the collection of qualitative data, including notes from attendance at project meetings, narratives, and scores compiled through application of the CFIR with project team members. Interview transcripts and notes from the document review will be compiled and reviewed by at least 2 mHealth team members to determine a preliminary coding framework using NVivo. This framework will constantly be reviewed, adapted, and reworked in an iterative manner so as to include data collected from each session and project team. This iterative process will also highlight instances that are unique to specific social and political contexts and that may require further discussion and clarification with project team members.

For the quantitative scoring data, *t* tests, clustering, or repeated measure analysis will be used based on the data to test the relationship between each of the factors and implementation success. By looking at how things were supposed to happen, we will tease out the gap between the ideal and actual result of the research. The gap between research conducted in a controlled setting and the implementation of an intervention or service into a “real-world” environment is often wide. Understanding the relationships between what was supposed to happen and what actually happened will highlight important considerations for implementation. For example, the study protocol might state that a clinician will follow up on all nonresponders with a telephone call. Six months into the study, the realization that most nonresponders are fine and simply “forgot” to respond may prompt a change to the procedure. Going forward, the clerical staff might now assume this role and will triage clinical questions to a nurse. The details outlining what actually happened will provide valuable insight into how the intervention can be integrated into future clinical care. This kind of detailed contextual information will be collected and analyzed across the different projects.

### Ethical Considerations

The study protocol, information and consent form, and questionnaires were approved by the University of British Columbia’s Clinical Research Ethics Board (H15-03478) and Behavioral Research Ethics Board (H16-00189), and the Amref Ethics and Scientific Review Committee (AMREF-ESRC P161/2015). Ethical approval will be renewed on an annual basis. Written informed consent will be sought from interview and focus group participants and those being observed and participating in project study meetings. Meeting participants can indicate to mHealth research team members conducting participant observation that they do not want their comments recorded or that specific topics of a sensitive nature should not be recorded. Those who decline participation will not be included in the study. As a form of member checking, participants will be given the opportunity to review draft reports/articles/summaries of our evaluation. All names will be changed to a participant-chosen pseudonym.

### Consent to Participate

Project stakeholders will be identified by each project team. Once a list of names for each project has been generated, the program manager or senior research fellow will email them a description of the study and invite them to participate in the focus group or interview. A copy of the consent form will be included in the email. If they decide they would like to participate, they will be asked to sign a copy of the consent form at the location of the focus group. To attend meetings of projects, the research manager or senior research fellow will ask permission of the principal investigator of each study. They will ask the principal investigator to email the team ahead of time, alerting them to their intention to attend the meeting, and will include the consent form in the email. At the meeting, they will provide a brief description of the study. All of those in attendance who would like to be involved and potentially contacted in the future to participate in an interview will be given a consent form and asked to sign. For those who do not want to participate, no mention of them will be made in the notes taken by the researcher in attendance.

### Confidentiality

To maintain participant confidentiality, all identifying information will be removed from questionnaires and study documents. Participants will be identified with a unique clinic identification number that is known only to a limited number of trained clinical and research personnel. Study information containing personal information, such as enrollment and informed consent forms, will be stored in locked filing cabinets offsite with limited access. All personal identifying information will be removed from interview transcriptions. Any information stored on computer databases will be password protected with limited access.

## Results

The development of the customized CFIR is completed and currently is in pilot testing. The initial results of the use of the tool in those 13 implementations will be available in 2019. Continuous conference and peer-reviewed publications will be published in the coming years.

## Discussion

### Principal Considerations

While there are many pilot studies and trials investigating the application of mHealth to improve adherence and other health outcome indicators, such as morbidity and mortality, there is a lack of sufficient evidence to inform the scale-up of mHealth programs in real-world settings. According to Luoto et al [41], ideal evidence for scale-up of mHealth programs includes efficacy (does it work?), effectiveness (does it work in a variety of populations and contexts?), and sustainability (cost effectiveness, demand, adaptation into health system). Following on from this, the overarching goal of our proposed research is, therefore, to close the gap in the evidence required to deliver an effective mHealth service to support patient care at scale in multiple global settings.

Our proposed implementation science study contains four important innovations. First, this study comprises 13 currently funded mHealth projects and programs. As outlined by Edwards et al [42], without attending to context and how it interacts with interventions, implementation of interventions are likely to fail or underperform. Hence our study, based in four countries across two continents, will provide robust, context-specific implementation science evidence to assist in moving the mHealth field forward.

Second, this proposed implementation science study will also compare barriers and facilitators to implementation of mHealth in both high-resource and low-resource settings. Sood et al [43] conducted a systematic review to compare different facilitators and barriers in developed and developing countries for successful electronic medical record implementation and use and found a major difference in barriers in those two settings. However, with respect to the implementation of mHealth interventions, this type of analysis is lacking and this study aims to fill this evidence gap. The results of this study therefore might advance the field of mHealth implementation science by examining predictors of implementation and sustainability as a function of intervention stage, which will give us implementation status‒based evidence to inform future implementation of similar interventions.

Third, our 13 mHealth projects across the different settings are at different stages. Some are starting, some are in the pilot phase, some are in the transition to scale phase, and some are already adopted as regular programs within the health system. This programmatic variety is a unique opportunity to compare facilitator and barrier factors based on the stage of the project. Additionally, we will be collecting data about the facilitator and barrier factors continuously every 6 months for each of the projects over 5-year period. This will give insight on how the implementation factors change as time advances and at difference phases of implementation. This will help us generate evidence on barrier and facilitator factors through the beginning, middle, and end of the project periods.

Last, this study will adapt an easy-to-use, pragmatic scoring scheme using the CFIR framework. Overall, this will be one of the first studies to perform cross-project identification of predictors of implementation and sustainability of mHealth in a global setting. After 3 years with substantial data from all 13 sites over time, we will be able to review and make recommendations to adjust and make any necessary improvements to this tool. Our goal is to develop a tool that can be used not only for this project, and by this research group, but by others seeking to implement digital health and global health innovations around the world.

### Strengths and Limitations

The proposed study is one of the first to conduct a cross-project examination of individual- and organizational-level predictors of mHealth implementation in global setting. Our menu-of-constructs approach, using the CFIR, is beneficial to frame our study and compare findings with others. However, like other different framework driven studies, there might be some factors that are beyond our capacity to capture. We mitigate this by including a suggestion box under each domain to catch factors that are out of the included constructs in the CFIR framework. The other novel aspect of our approach is the concept of scoring for each of the constructs. However, the scoring will be based on individual perception, which might be biased. We mitigate this by including 5 participants on each of the projects. The other limitation to note is the comparison challenge between sites as different implementations are at different stages and different disease domains. We will mitigate this limitation by collecting data continuously during the entire phase of the project and perform the comparison analysis when the projects are in a similar stage.

### Conclusions

Many interventions found to be effective in health services research studies fail to translate into meaningful patient care outcomes across multiple contexts in settings outside the RCT environment. The conventional wisdom is that two-thirds of organizations’ efforts to implement change fail. In mHealth, there is no concrete evidence on the level of success and failure and why that happens. Our implementation science evaluation will fill a major void in this rapidly emerging field by identifying facilitators and barriers of mHealth success or failure. The evidence generated will help us document in a structured way the lessons learned by each project team and will identify how this information can inform the scale-up of mHealth interventions across diverse settings in Africa and North America, and ultimately beyond.

## Appendix

Multimedia Appendix 1 CIHR peer-review report.

## Abbreviations

ART: antiretroviral therapy
cDOT: community-based directly observed antiretroviral therapy
CIHR: Canadian Institute of Health Research
CFIR: Consolidated Framework for Implementation Research
UNAIDS: Joint United Nations Programme on HIV and AIDS
LTBI: latent tuberculosis infection
mHealth: mobile health
SMS: short message service
RCT: randomized controlled trial
TB: tuberculosis
WHO: World Health Organization
