# Supplementary material for: Identifying Barriers and Facilitators of 13 mHealth Projects in North America and Africa: Protocol for a 5-Year Implementation Science Study
Source: JMIR Res Protoc. 2018 Jul 3;7(7):e162. doi: 10.2196/resprot.9633 (PMC6053607; doi:10.2196/resprot.9633)
Supplement: Multimedia Appendix 1 [file resprot_v7i7e162_app1.pdf]

**Review Type / Type d'évaluation:**	Committee Member 1/Membre de comité 1
**Name of Applicant / Nom du chercheur:**	LESTER,Richard
**Application No. / Numéro de demande:**	332590
**Agency / Agence:**	CIHR/IRSC
**Competition / Concours:**	Foundation Scheme/Volet Fondation
**Committee / Comité:**	Foundation Scheme/Volet Fondation
**Title / Titre:**	Evidence to action for Canadian and global mobile health (mHealth) communication to promote patient engagement in care: a rigorous implementation science approach

Caliber of the Applicant(s)/Calibre des candidats

Criterion/Critère: Leadership/Leadership

Rating/Cote: E

Strengths/Forces: - track record of successfully developing collaborative initiatives that span countries, with the goal of improving international health
- has been identified as an "leader in global mHealth innovation" - evidence of international influence
- impact for stage of career shows potential for future development

Weaknesses/Faiblesses: - limited leadership positions beyond the research collaborations

Criterion/Critère: Significance of Contribution/Importance des contributions

Rating/Cote: O

Strengths/Forces: - prior research has had tangible benefits on recommendations for treating HIV and other diseases, both abroad and locally
- research has been highlighted both by the scientific community and the lay press
- good publication record, including higher impact medical journals
- several grad students supervised as primary supervisor

Weaknesses/Faiblesses: - none obvious, except relatively early in scientific career

Criterion/Critère: Productivity/Productivité

Rating/Cote: E

Strengths/Forces: - successful in obtaining some large grants to support ongoing research
- high impact publications, and evidence of ongoing/sustained productivity evidenced by number of publications
- engaged in mentorship and supervision

Weaknesses/Faiblesses: none

Vision and Program Direction/Vision et direction du programme

Criterion/Critère: Vision and Program Direction/Vision et direction du programme

Rating/Cote: G

Strengths/Forces: - overall goals of this proposed research are important

Weaknesses/Faiblesses: - limited information about actual methodologies that will be applied

**Review Type / Type d'évaluation:**	Committee Member 2/Membre de comité 2
**Name of Applicant / Nom du chercheur:**	LESTER,Richard
**Application No. / Numéro de demande:**	332590
**Agency / Agence:**	CIHR/IRSC
**Competition / Concours:**	Foundation Scheme/Volet Fondation
**Committee / Comité:**	Foundation Scheme/Volet Fondation
**Title / Titre:**	Evidence to action for Canadian and global mobile health (mHealth) communication to promote patient engagement in care: a rigorous implementation science approach

Caliber of the Applicant(s)/Calibre des candidats

Criterion/Critère: Leadership/Leadership

Rating/Cote: O+

Strengths/Forces: Has led international studies, Director of the UBC Neglected Global Diseases Initiative. Led three international meetings that stimulated new research collaborations and generated concrete outputs, such as partnership agreements and funding applications. Invited to participate as a 'leader in global mHealth innovation' in meetings hosted in Canada, the US, and China, and by organizations such as the World Health Organization and the United Nations Foundation.

Weaknesses/Faiblesses: None.

Criterion/Critère: Significance of Contribution/Importance des contributions

Rating/Cote: O++

Strengths/Forces: Very high impact work with research featured in top journals, and has shaped international guidelines for HIV.

Weaknesses/Faiblesses: None.

Criterion/Critère: Productivity/Productivité

Rating/Cote: O++

Strengths/Forces: High impact publications and clear ability to secure funding from many sources.

Weaknesses/Faiblesses: None.

Vision and Program Direction/Vision et direction du programme

Criterion/Critère: Vision and Program Direction/Vision et direction du programme

Rating/Cote: O+

Strengths/Forces: Vision for program builds clearly from prior research. Innovative and focused.

Weaknesses/Faiblesses: None.

**Review Type / Type d'évaluation:**	Committee Member 3/Membre de comité 3
**Name of Applicant / Nom du chercheur:**	LESTER,Richard
**Application No. / Numéro de demande:**	332590
**Agency / Agence:**	CIHR/IRSC
**Competition / Concours:**	Foundation Scheme/Volet Fondation
**Committee / Comité:**	Foundation Scheme/Volet Fondation
**Title / Titre:**	Evidence to action for Canadian and global mobile health (mHealth) communication to promote patient engagement in care: a rigorous implementation science approach

Caliber of the Applicant(s)/Calibre des candidats

Criterion/Critère: Leadership/Leadership

Rating/Cote: O+

Strengths/Forces: The applicant has demonstrated leadership in his field, inspiring others and mobilizing communities in advancing the direction of a field. For example, when in Kenya, he created a new program area of research using mobile phones to improve HIV/AIDS care. This involved conceptualizing the idea, engaging multidisciplinary teams, and securing research funding. Since then, he has led multi-site Canadian and international studies, including a United States NIH trial to determine whether mHealth can
improve retention in HIV care in a Kenyan urban slum, among Canadians with latent tuberculosis infection (G9), and Grand Challenges Canada projects to scale-up
the intervention in Kenya. This research has inspired others to apply the mHealth service, such as testing the mHealth intervention among at-risk Aboriginal youth and adapting the intervention for those with asthma.

The applicant has successfully established, resourced and led programs of research: three international meetings that stimulated new research collaborations and generated concrete outputs, such as partnership agreements and funding applications; invited to participate as a ‘leader in global mHealth innovation’ in meetings hosted in Canada, the US, and China, and by organizations such WHO and UN Foundation; nomination as Director of the UBC Neglected Global Diseases Initiative provides greater scope for health innovation leadership and growth.

Weaknesses/Faiblesses: N/A

Criterion/Critère: Significance of Contribution/Importance des contributions

Rating/Cote: O++

Strengths/Forces: The applicant has significantly advanced knowledge, research, health care, health systems and/or health outcomes: conducted in influenza and HIV pathogenesis are recognized as contributions to their fields; as BCCDC’s Medical Head of STI/HIV, he influenced provincial and national gonorrhea treatment guidelines, which resulted in improving trends in BC; as a pioneer in the growing field of mHealth, his research directly influenced International Association of Providers of AIDS Care (IAPAC) and WHO guidelines on the use of text messaging to support HIV care; he brought our mHealth program to North America from Kenya as a form of ‘reverse innovation’, and the program continues be an example of how mobile communication can be used to improve the health of Canadians. Knowledge translation strategies and activities are an integral part of his research, and include a number of policy letters in top-tier medical journals. His research has also generated significant public interest, and has appeared in international news and scientific media. The applicant gave several keynote addresses at international conferences, and one of his current trials was lauded as an example to others by the Director of the US NIH.

The applicant has engaged, trained, and/or launched the careers paths of promising individuals in research and/or other health-related non-academic fields. For example, his work has attracted students and trainees from medicine and other disciplines. He is currently supervising five doctoral students and two post-doctoral researchers. Several of his graduate students and residents have been the recipients of highly competitive funding, including CIHR doctoral awards and clinical

investigator awards.

Weaknesses/Faiblesses: N/A

Criterion/Critère: Productivity/Productivité

Rating/Cote: O++

Strengths/Forces: The applicant demonstrated an outstanding level of research outputs, based on prior work, and the applicant's previous work generated high quality research outputs.
For example, the applicant planned a randomized controlled trial to rigorously answer questions on effectiveness of the mHealth idea in Kenya. He successfully lobbied for funding to use viral load testing (not widely available locally at the time) to test innovation against objective biological outcomes. This trial was published as Editor's Choice in the Lancet; the trial was the earliest evidence in the field and was later graded as the highest level of-quality of evidence by the Cochrane Review. Subsequent studies based on trial data included an in-depth investigation of patient-clinician communication and an essential cost-effectiveness investigation of the intervention.

The applicant subsequently have secured $2,307,840 in recent operating grants to broaden his mHealth research program to investigate the effectiveness of mHealth in new geographical areas and in other patient populations. The applicant is currently conducting trials of the intervention among patients in earlier stages of HIV care, with latent tuberculosis infection, in those with asthma, and in other geographic areas. Where they already have evidence of effectiveness, they have launched projects in implementation science and transition to scale. The applicant strives not only to create and test new innovations, but to fill many of the knowledge gaps in the field. To translate evidence to action, the applicant uses a range of knowledge translation strategies to actively engage policy makers and the public through all stages of the research process.

Weaknesses/Faiblesses: N/A

Vision and Program Direction/Vision et direction du programme

Criterion/Critère: Vision and Program Direction/Vision et direction du programme

Rating/Cote: O++

Strengths/Forces: The vision of the applicant's research program is to expand leadership in mHealth and develop a world-class global health research and innovation program, focusing on improving the care of remote and hard-to-reach populations. The applicant aims to make global health innovation the hallmark of his contribution to the global academic arena, with the ultimate aim to positively impact health care and outcomes. The applicant's goal is to fill many of the critical gaps in knowledge on why, how, when, where and for whom mHealth can best improve patient health outcomes. The applicant will apply lessons learned to other clinical areas of global
health to benefit populations in Canada and abroad. Over the next five years, the applicant will scale up the WelTel text-messaging system to provincial, national, and international sites, and use rigorous scientific methods to evaluate processes and outcomes. To facilitate this, the applicant has developed an academic path linked with social enterprise partnerships including the non-profit society and WelTel software company.

The applicant will fulfilling the following objectives:

1) Determine the individual, population, health system, and health environment factors associated with the successful uptake, effectiveness, and cost-effectiveness of the WelTel mHealth intervention in Canada and Africa, using an established implementation science framework.

2) Consolidate multiple mHealth research studies into a growing database that allows cross-comparisons of factors and outcomes to answer critical research questions in the field.

3) Use a broad range of integrated knowledge translation activities to synthesize and disseminate findings, and to exchange knowledge with others.

Weaknesses/Faiblesses: N/A

**Review Type / Type d'évaluation:**	Committee Member 4/Membre de comité 4
**Name of Applicant / Nom du chercheur:**	LESTER,Richard
**Application No. / Numéro de demande:**	332590
**Agency / Agence:**	CIHR/IRSC
**Competition / Concours:**	Foundation Scheme/Volet Fondation
**Committee / Comité:**	Foundation Scheme/Volet Fondation
**Title / Titre:**	Evidence to action for Canadian and global mobile health (mHealth) communication to promote patient engagement in care: a rigorous implementation science approach

Caliber of the Applicant(s)/Calibre des candidats

Criterion/Critère: Leadership/Leadership

Rating/Cote: O+

Strengths/Forces: strong evidence of international leadership and impact; incoming director of UBC neglected global diseases initiative

Weaknesses/Faiblesses: lack of consideration of academic leadership (e.g., advancements to the discipline); would like to see discussion of how lasting international partnerships are or have been

Criterion/Critère: Significance of Contribution/Importance des contributions

Rating/Cote: O++

Strengths/Forces: has influenced WHO guidelines; research has had a demonstrable impact in the field of mHealth; has attracted media coverage

Weaknesses/Faiblesses: would like to see a broader description of knowledge translation strategies used

Criterion/Critère: Productivity/Productivité

Rating/Cote: O++

Strengths/Forces: continuous record of research funding;

Weaknesses/Faiblesses: would like to see a quantative summary of publications as well

Vision and Program Direction/Vision et direction du programme

Criterion/Critère: Vision and Program Direction/Vision et direction du programme

Rating/Cote: E++

Strengths/Forces: logical extension of the applicants' existing research

Weaknesses/Faiblesses: clarification is needed on who the "our" and "we" being referred to are; first postdoctoral placement was started in 2002 so it's not clear why applicant is referring to themselves as a 'new investigator' in this application

**Review Type / Type d'évaluation:**	Committee Member 5/Membre de comité 5
**Name of Applicant / Nom du chercheur:**	LESTER,Richard
**Application No. / Numéro de demande:**	332590
**Agency / Agence:**	CIHR/IRSC
**Competition / Concours:**	Foundation Scheme/Volet Fondation
**Committee / Comité:**	Foundation Scheme/Volet Fondation
**Title / Titre:**	Evidence to action for Canadian and global mobile health (mHealth) communication to promote patient engagement in care: a rigorous implementation science approach

Caliber of the Applicant(s)/Calibre des candidats

Criterion/Critère: Leadership/Leadership

Rating/Cote: E+

Strengths/Forces: Excellent demonstration of effectively leading research teams, securing resources, ensuring effective collaboration; evidence of advancing direction of the field in terms of mobile technologies to improve health.

Weaknesses/Faiblesses: Increasing evidence of being recognized in this field nationally and internationally in terms of invited presentations at research conferences.

Criterion/Critère: Significance of Contribution/Importance des contributions

Rating/Cote: E++

Strengths/Forces: Excellent documentation of contribution to knowledge, research, health care, health systems. Evidence of effective mentoring of junior researchers.

Weaknesses/Faiblesses: Given the level of funding, I would have expected more publications as lead or senior author in top-tier journals

Criterion/Critère: Productivity/Productivité

Rating/Cote: O

Strengths/Forces: Very successful in securing large research grants (>2.5M currently active grants as PI)

Weaknesses/Faiblesses: There may be room for improvement in terms of more recent first or senior author publications in top-tier journals; total 39 publications in the past 10 years - this number will likely increase given the high level of funding.

Vision and Program Direction/Vision et direction du programme

Criterion/Critère: Vision and Program Direction/Vision et direction du programme

Rating/Cote: E+

Strengths/Forces: Vision is focused on improving care of remote and hard-to-reach populations. This is well-defined and logical progression in the applicant's career path.

Weaknesses/Faiblesses: I am not convinced about the vision and the three stated objectives being sufficiently forward-looking, creative, and appropriately ambitious.
Relevance of m-health to Canadian population? Generalizability from the HIV and developing country setting to other settings?
Appropriate collaborative ties with specialists in implementation science and knowledge translation?

**Review Type / Type d'évaluation:**	Committee Member 1/Membre de comité 1
**Name of Applicant / Nom du chercheur:**	LESTER,Richard
**Application No. / Numéro de demande:**	332590
**Agency / Agence:**	CIHR/IRSC
**Competition / Concours:**	Foundation Scheme/Volet Fondation
**Committee / Comité:**	Foundation Scheme/Volet Fondation
**Title / Titre:**	Evidence to action for Canadian and global mobile health (mHealth) communication to promote patient engagement in care: a rigorous implementation science approach

Quality of the Program/Qualité du programme

Criterion/Critère: Research Concept/Idée de recherche

Rating/Cote: O

Strengths/Forces: Adherence to medication and retention in care are very important factors in determining the effectiveness of health care. The applicant has designed a series of studies to further examine how mobile phone technology can assist in this regard. The proposal clearly builds on his previous experience in conducting RCTs to examine the use of this technology. Furthermore it builds on efforts to expand the impact of this research beyond scientists to facilitate a massive scale up of this technology. The outputs from this program are potentially quite important, since they are designed to eventually have population-level impact.

Weaknesses/Faiblesses: The breadth of the setting and types of conditions under study here appear a bit disparate. However, this could also be seen as a strength. Of the objectives listed in the proposal, only objective 1 is truly a research objective.

Criterion/Critère: Research Approach/Approche de recherche

Rating/Cote: E+

Strengths/Forces: A clear series of studies are outlined, all designed to test SMS technology in a variety of settings. The studies will use similar data collection tools so as to allow for comparisons across studies.

Weaknesses/Faiblesses: The value of the cross-study comparison is not clear. It would seem that each intervention should be compared with other potential interventions which could also be used to increase adherence for each of the conditions of interest.

Quality of the Expertise, Experience, and Resources/Qualité de l'expertise, de l'expérience et des ressources

Criterion/Critère: Expertise/Expertise

Rating/Cote: O

Strengths/Forces: The applicant is an Assistant Professor in the Department of Medicine at UBC. He has recently been appointed to a Full-time global health academic position. Interestingly he has also founded an NGO designed specifically to promote the use of SMS technology for health. He has received two Grand Challenges grants, including a current grant for $1M. He has published 44 research papers.

Weaknesses/Faiblesses: None.

Criterion/Critère: Mentorship and Training/Mentorat et formation

Rating/Cote: E

Strengths/Forces: He has supervised 9 graduate students and 2 post-docs.

Weaknesses/Faiblesses: None of his previous trainees are currently in professional positions. However, until recently, the applicant had a clinical appointment which likely limited his ability to supervise grad students. The opportunities to expand mentoring and supervision are not well discussed, especially for trainees from outside Canada.

Quality of Support Environment/Qualité de l’environnement de soutien

**Review Type / Type d'évaluation:**	Committee Member 1/Membre de comité 1
**Name of Applicant / Nom du chercheur:**	LESTER,Richard
**Application No. / Numéro de demande:**	332590
**Agency / Agence:**	CIHR/IRSC
**Competition / Concours:**	Foundation Scheme/Volet Fondation
**Committee / Comité:**	Foundation Scheme/Volet Fondation
**Title / Titre:**	Evidence to action for Canadian and global mobile health (mHealth) communication to promote patient engagement in care: a rigorous implementation science approach

Criterion/Critère: Quality of Support Environment/Qualité de l'environnement de soutien

Rating/Cote: E++

Strengths/Forces: Good facilities available at UBC and in Kenya. The development of two NGOs to facilitate the development of the mhealth technology is a clear strength.

Weaknesses/Faiblesses: No discussion of data management or analysis support, although one imagines that these areas must be in place already to facilitate the applicants ongoing work.

**Review Type / Type d'évaluation:**	Committee Member 2/Membre de comité 2
**Name of Applicant / Nom du chercheur:**	LESTER,Richard
**Application No. / Numéro de demande:**	332590
**Agency / Agence:**	CIHR/IRSC
**Competition / Concours:**	Foundation Scheme/Volet Fondation
**Committee / Comité:**	Foundation Scheme/Volet Fondation
**Title / Titre:**	Evidence to action for Canadian and global mobile health (mHealth) communication to promote patient engagement in care: a rigorous implementation science approach

Quality of the Program/Qualité du programme

Criterion/Critère: Research Concept/Idée de recherche

Rating/Cote: O

Strengths/Forces: There is solid justification of the concept from a sound evidence base. The evidence for using the approach is proven for reaching those most vulnerable who have the least access to the health system.
The goals and objectives are clearly outlined and there is cohesion across the different facets of the program.
Importantly, there is already sound evidence that the program works in one disease (and setting), and randomised controlled trials are already underway to test the efficacy in other disease conditions (e.g. TB and asthma). In addition, there is already funding for scale-up of HIV in Kenya (one of the program sites).
There is considerable strength in the potential to value-add by integrating the current studies into a larger database. This will enable greater power to examine factors associated with outcomes and determine differences across settings.
Potential outputs are significant, and include likelihood of high quality publications and translation to an improved method of health care.

Weaknesses/Faiblesses: There is quite a diverse range of diseases being studied. While this has the potential to greatly enhance the productivity of the team, it also has the potential to distract from the overarching goal of determining scalability of the program.

Criterion/Critère: Research Approach/Approche de recherche

Rating/Cote: O

Strengths/Forces: The evaluation of implementation of the program is based on a high quality and adaptable framework that includes both evaluation of endpoints as well as formative evaluations to determine effectiveness in differing contexts.
There are clear processes for measuring success including training, outputs (e.g. publications), uptake by stakeholders, health outcomes and improvements in the health system. The challenge of coordinating independent studies into a single program is mitigated by ensuring appropriate methods are in place to effectively consolidate the database, as well as ensuring that the evaluations are broad.
It is likely that the approach will enable the investigators to deliver on the program

Weaknesses/Faiblesses: Flexibility is somewhat restrained by the nature of the investigation, although it is possible that evaluation may identify some novel component that requires further investigation. The applicants don’t appear to have considered that there may be avenues to enable the program to evolve.

Little mention is made of the strategies to involve and engage stakeholders, despite this being a critical element of implementation science

Quality of the Expertise, Experience, and Resources/Qualité de l'expertise, de l'expérience et des ressources

Criterion/Critère: Expertise/Expertise

Rating/Cote: E

Strengths/Forces: The program leader has the capacity and expertise to led this program of research. He has instigated many of the research studies which underpin this application. He has further co-founded a non-profit organisation designed to enhance scalability of their mhealth research. He has also published some influential results on the use of mhealth, and his research has been instrumental in modifying guidelines.

Involvement of government and non-government organisations is also a strength, although the level of engagement is unclear.

There is also inclusion of a multidisciplinary team of program experts with expertise in infectious diseases, medication adherence, and a social scientist. There is also significant support, mainly in-kind, from program partners that demonstrates considerable commitment by these partners.

Weaknesses/Faiblesses: While there is significant expertise in infectious diseases there appears to be a lack of inclusion (apart from trainees) of expertise in evaluation, implementation science, assessment of cost-effectiveness and scalability. These are critical components of such a program of research. While there is some developing expertise in this area from trainees, more senior direction is lacking.

Criterion/Critère: Mentorship and Training/Mentorat et formation

Rating/Cote: O++

Strengths/Forces: The program for mentoring and training includes both a formal and informal component. This includes training from external partners as well as internally. There are systems in place to encourage independence as well as support to attend external training courses.

A significant training and mentoring opportunity is provided by the program leader who directs some invitations to present his work to mentors and trainees who are at an adequate lev el to undertake this successfully. The program leader should be congratulated for implementing such an approach as these opportunities enable junior colleagues to shine!

There are also opportunities for junior colleagues to engage with industry and this enhances non-academic career progression as well as academic career progression.

Mitigation of challenges to multidisciplinary training is undertaken by harnessing a broad network of expertise to supervise, mentor, and otherwise engage with trainees.

Success and progress is measured by presentations, publications and awards. This is a comprehensive program of training and will ensure preparation of trainees for their chosen careers.

Weaknesses/Faiblesses: None.

Quality of Support Environment/Qualité de l’environnement de soutien

Criterion/Critère: Quality of Support Environment/Qualité de l’environnement de soutien

Rating/Cote: O

Strengths/Forces: All the necessary infrastructure is provided including personnel. There is access to high quality facilities that support the program of research, including videoconferencing.

There is significant and growing infrastructure internationally including Amref Health Africa and the University of Nairobi. Infrastructure (both hardware and software) is in place to support the research program in Kenya.

The applicants have developed their own software company to develop a sustainable information technology platform that can also support scale-up. There is also a considerable network that supports the program, as well as evidence of media

access (from previous media reports).

Weaknesses/Faiblesses: None

**Review Type / Type d'évaluation:**	Committee Member 3/Membre de comité 3
**Name of Applicant / Nom du chercheur:**	LESTER,Richard
**Application No. / Numéro de demande:**	332590
**Agency / Agence:**	CIHR/IRSC
**Competition / Concours:**	Foundation Scheme/Volet Fondation
**Committee / Comité:**	Foundation Scheme/Volet Fondation
**Title / Titre:**	Evidence to action for Canadian and global mobile health (mHealth) communication to promote patient engagement in care: a rigorous implementation science approach

Quality of the Program/Qualité du programme

Criterion/Critère: Research Concept/Idée de recherche

Rating/Cote: O++

Strengths/Forces: - one of the every best in this competition I have read - focused, builds and expands upon a solid foundation, innovative, and a pleasure to read.

Weaknesses/Faiblesses: - none

Criterion/Critère: Research Approach/Approche de recherche

Rating/Cote: O+

Strengths/Forces: - suitably high level, allowing for flexibility.

Weaknesses/Faiblesses: - I would have liked to see how the numerous studies link and feed off each other, and more specific details, to ensure that they could be executed as planned.
- nonetheless, the PI has a tremendous track record of funding for these studies, so that is evidence in itself of their quality.
- the PI is cognisant of the challenge of keeping so many balls in the air at the same time, but as he builds a team, and transitions to a more academic role, he should be able to carry this off.

Quality of the Expertise, Experience, and Resources/Qualité de l'expertise, de l'expérience et des ressources

Criterion/Critère: Expertise/Expertise

Rating/Cote: O++

Strengths/Forces: - absolutely fantastic track record, and a very strong team, both domestically and internationally.

Weaknesses/Faiblesses: - none that I can see.

Criterion/Critère: Mentorship and Training/Mentorat et formation

Rating/Cote: O++

Strengths/Forces: - the young investigator has received the most exceptional support.
- has a well rounded plan for mentorship and training - already has had tremendous success in "handing off" projects to others.

Weaknesses/Faiblesses: - the only question I want to know is - who is the PI's mentor? It would be wise to have a senior advisor as he will very quickly need some sage advice and mentoring himself as his research program expands.

Quality of Support Environment/Qualité de l’environnement de soutien

Criterion/Critère: Quality of Support Environment/Qualité de l’environnement de soutien

Rating/Cote: O++

Strengths/Forces: - exceptionally well supported, and well connected in Canada and Africa.

Weaknesses/Faiblesses: - none.

**Review Type / Type d'évaluation:**	Committee Member 4/Membre de comité 4
**Name of Applicant / Nom du chercheur:**	LESTER,Richard
**Application No. / Numéro de demande:**	332590
**Agency / Agence:**	CIHR/IRSC
**Competition / Concours:**	Foundation Scheme/Volet Fondation
**Committee / Comité:**	Foundation Scheme/Volet Fondation
**Title / Titre:**	Evidence to action for Canadian and global mobile health (mHealth) communication to promote patient engagement in care: a rigorous implementation science approach

Quality of the Program/Qualité du programme

Criterion/Critère: Research Concept/Idée de recherche

Rating/Cote: O+

Strengths/Forces: Summary Assessment:

The investigator seeks to determine the impact of mHealth communication to promote patient engagement. The research concept is excellent. It is comparatively novel with the potential to have real impact on health outcomes. The investigator provides a convincing rationale for the importance of utilizing text messaging as a mechanism to improve patient adherence. He has demonstrated in an RCT the impact such a mechanism can have on actual patient outcomes such as viral load. He has obtained funding for the extension of the work to other diseases. Given the focus on the potential value of this area, the field deserves study to determine if mHealth interventions are truly effective.

Specific Assessment

Are the goals and objectives of the proposed program well-defined and well-articulated? Yes, to gather evidence to develop an effective mHealth intervention. A more neutral approach may have been preferred examining if mHealth is truly useful and in which settings it is best suited.

Is there conceptual coherence within the program of research? Yes. The program involves a serious of mostly funded studies examining the potential value of mHealth interventions.

Are the potential program outputs significant? Are they likely to significantly advance health-related knowledge and/or its translation into improved health care, health systems and/or health outcomes? This was somewhat more difficult to assess given the inability to discuss the specific projects in detail. However, it appears that the outcomes being examined include objective health outcomes.

Weaknesses/Faiblesses: The proposal does seem to imply that mHealth will work and its a matter of determining how to implement it. A more balanced approach would have been preferrable.

It was difficult to assess the impact of the specific studies that were proposed.

Criterion/Critère: Research Approach/Approche de recherche

Rating/Cote: O

Strengths/Forces: Summary Assessment

The investigator briefly describes 8 projects, six of which are funded. At least 4 of these are RCT's. Unfortunately, not much detail is provided about the methods for any of the studies so it is difficult to evaluate whether the research approach is appropriate. However, as many of these have been funded by granting agencies such as CIHR, Grand Challenges Canada, one can assume that the methodology is sufficiently rigorous. The research approach described also seems to be sufficient to achieve the overall goals described in the proposal and includes, effectiveness research, cost-effectiveness research, qualitative research as well as the creation of databases for further analyses.

Specific Assessment

Is the approach appropriate to deliver on the proposed program objectives? Yes. The network of studies proposed should generate a valuable database that provides information on the utility of mHealth.
Does the approach allow for flexibility in direction as the program evolves? Yes. The program provides a framework for which the result of the studies will feed. However, it is uncertain how the investigator would proceed if the studies determine no value or potentially harm associated with the intervention.
Does the approach include a high level description of how progress and success will be measured? Yes. And this includes an assessment of hard health outcomes.
Does the approach include a plan for identifying potential challenges and applying appropriate mitigation strategies? Somewhat. It remains uncertain how the investigator would proceed if mHealth interventions fail.

Weaknesses/Faiblesses: None of the projects is described in sufficient detail to have a full understanding of the adequacy of the research approach. The integration of the qualitative component of the program with the quantitative was not clear. It remains unclear as to how the investigator would proceed if mHealth interventions fail or are found to be harmful. As the investigator is the creator of the technology a mechanism to mitigate conflict of interest in the research could be described.

Quality of the Expertise, Experience, and Resources/Qualité de l'expertise, de l'expérience et des ressources

Criterion/Critère: Expertise/Expertise

Rating/Cote: E

Strengths/Forces: Summary assessment:

The investigator has successfully built an innovative program and a novel technology. He has published a high impact paper in the Lancet that demonstrated the impact of this technology and obtained funding for several other projects. The investigator has provided advice on the content area he studying internationally. The team of collaborators is strong and complements the program well.

Specific Assessment:

Does the applicant(s) have the appropriate expertise and relevant experience to lead and manage the proposed program of research, considering its objectives and scope? Yes. However, it would bring more confidence if further evidence of success in some of the other mHealth projects was available.
Is there an appropriate complement and level of engagement and/or commitment from key Program Expert(s)? Yes. Although they are all fairly local.

Weaknesses/Faiblesses: The lead investigator has a fairly modest publication record compared to others in this competition. It appears as though he may have only recently emabarked upon an academic focus to his career which might explain this. It would bring more confidence to have also seen more outputs from some other WelTel projects. Given the potential international focus of the program a less local group of program experts would have been of value.

Criterion/Critère: Mentorship and Training/Mentorat et formation

Rating/Cote: G

Strengths/Forces: Summary Assessment:

The proposal identifies 4 individuals who will be trained by the program a post-doctoral fellow and three PhD students. The investigators role in supervising these candidates in some instances is not clear.

Specific Assessment:

Does the research program include a comprehensive mentorship and training plan for building capacity and positioning students, trainees, knowledge-users, emerging scholars and/or new/early career investigators for successful research careers and/or other career paths in non-academic health-related fields? Somewhat. The training portion is comparatively less developed to other applications and to other components of this application and doesn't seem to have a clear infrastructure to continue to produce trainees.

Does the proposed plan demonstrate an appropriate and innovative approach for meeting its objectives, in relation to the program of research and the research field? Somewhat. Not a lot of detail is provided on the trainee component of the program

Does the plan include a strategy for identifying and mitigating potential challenges? Somewhat. Not many challenges are identified.

Weaknesses/Faiblesses: The roles of the investigator in training these individuals is unclear. The track record in supervising individuals is unclear. The actual infrastructure for training individuals needs more description.

Quality of Support Environment/Qualité de l'environnement de soutien

Criterion/Critère: Quality of Support Environment/Qualité de l'environnement de soutien

Rating/Cote: O

Strengths/Forces: Summary Assessment

The investigator has created an impressive infrastructure and extensive relationships to both support the research, assist with evaluation and translation into practice.

Specific Assessment

Is the described environment(s) appropriate to enable the conduct of the program of research and to manage and deliver on the objectives and key components of the proposed research program (e.g., research, knowledge translation, mentoring/training) through the provision of, or access to, the required infrastructure, such as:

Physical infrastructure (and/or other types of infrastructure such as consortia professional networks etc.); Yes. The investigator has developed the technology and the program to implement the research.

Support personnel; Sufficient

Equipment: Yes. Includes a software company

Specialized facilities: As above

Supplies: Not applicable.

Weaknesses/Faiblesses: None

**Review Type / Type d'évaluation:**	Committee Member 5/Membre de comité 5
**Name of Applicant / Nom du chercheur:**	LESTER,Richard
**Application No. / Numéro de demande:**	332590
**Agency / Agence:**	CIHR/IRSC
**Competition / Concours:**	Foundation Scheme/Volet Fondation
**Committee / Comité:**	Foundation Scheme/Volet Fondation
**Title / Titre:**	Evidence to action for Canadian and global mobile health (mHealth) communication to promote patient engagement in care: a rigorous implementation science approach

Quality of the Program/Qualité du programme

Criterion/Critère: Research Concept/Idée de recherche

Rating/Cote: E++

Strengths/Forces: This program intends to develop the evidence required to deliver an effective mobile health service to support patient care in multiple global settings.

In previous works, the applicants have developed and implemented the wel tel service. They have demosntratedin a trial that that interactive text-messaging between health providers and patients taking antiretroviral therapy for HIV significantly improved health outcomes.

The objectives of the new prooposed program are clearly stated and show a strong potential to develop new knowledge and inform the application of the WelTel service to several common chronic disease management settings in diverse contexts.The new proposed program is designed to undertake a comprehensive implementation science evaluation and to determine the individual, population, health system, and health environment factors associated with the successful uptake and effectiveness of the WelTel mHealth intervention in Canada and Africa.

The proposal builds upon a set of 8 projects, whose 6 are already funded.

Weaknesses/Faiblesses: The proposal builds upon a set of 8 projects, but what is clearly missing is to better show, in using the proposed conceptual framework, how the diverse projects are articulated and complement each other to build new knowledge.

Criterion/Critère: Research Approach/Approche de recherche

Rating/Cote: G

Strengths/Forces: Well structured program with a set of 8 projects

The Consolidated Framework for Implementation Research is proposed to guide the research.

Weaknesses/Faiblesses: The information given is too vague and does not provide sufficient indications on how the Consolidated Framework for Implementation Research will be operationalized troughout the diverse projects.

The applicants have a strong record on clinical trial, but the information provided is quite sketchy and is not sufficient to really assess the scientific quality of the proposal.

Quality of the Expertise, Experience, and Resources/Qualité de l'expertise, de l'expérience et des ressources

Criterion/Critère: Expertise/Expertise

Rating/Cote: E+

Strengths/Forces: The program leader has a strong funding history and a quite good record of publications

He has experience and expertise as concept development leader and has been leading several previous studies related to mobile health service. He has also founded the WelTel International mHealth Society.

The program will draw on a series of international partners that have established records of collaboration with the program leader.

The program will also draw on a solid team gathering local and international experts, knowledge users, clinicians, scientists, academic trainees, government policy makers, community leaders, etc.

Weaknesses/Faiblesses: A major gap is a lack of indications on mechanisms and structure that will be used to ensure active involvement of all members of such large team.

Criterion/Critère: Mentorship and Training/Mentorat et formation

Rating/Cote: G

Strengths/Forces: The program leader has previously supervised several undergraduates and is currently supervising five doctoral students and two post-doctoral researchers.
The applicants have clearly described their current academic environment which is highly supportive to training.

Weaknesses/Faiblesses: Although the applicants show that the program will be located in an excellent traning environment, they have not clearly shown how the program will take advantage of this environment to build new capacities. They have not presented a clear plan of capacity building, with explcit targets

Quality of Support Environment/Qualité de l'environnement de soutien

Criterion/Critère: Quality of Support Environment/Qualité de l'environnement de soutien

Rating/Cote: O+

Strengths/Forces: The program leader is the Director of the UBC Neglected Global Diseases Initiative, a trans-faculty and trans disciplinary initiative, which gives him access to the administrative leadership of UBC, a network of scientists and area leaders, as well as a range of material and support resources.
Internationally, the program may draw on the infrastructure of support previously built by the applicants and partnerships with international organizations and the University of Nairobi. The WelTel International mHealth Society, a BC-registered non-profit organization with a registered branch in Kenya and WelTel Inc., a software company, are other resources that will contribute to support the program

Weaknesses/Faiblesses: More information could be given on the linkages with other intellectual resources and research centres available in the program environment
